# Soil pH Amelioration Synergizes Carbon Accrual with Yield Gains Under Organic Substitution

**DOI:** 10.1002/advs.78051

**Published:** 2026-09-28

**Authors:** Xunzhuo Dong, Yanzhong Yao, Bingbing Han, Yunyao Zhong, Benyi Li, Mengfei Li, Yunchao Dai, Zhaolei Li

**Affiliations:** ^1^ State Key Laboratory of Soil and Water Conservation and Desertification Control College of Natural Resources and Environment Northwest A&F University Yangling China; ^2^ Interdisciplinary Research Center For Agriculture Green Development in Yangtze River Basin College of Resources and Environment Southwest University Chongqing China

**Keywords:** independent contribution, organic substitution, pH amelioration, soil organic carbon accrual, yield gain

## Abstract

Soil pH management underpins sustainable agriculture globally, yet the intrinsic effects of pH change on soil organic carbon (SOC) accrual and crop yields remain obscured by concomitant shifts in other soil properties. Here, using two complementary global field datasets, we disentangle pH‐specific contributions to SOC accrual and yield gains under organic substitution regimes (partial replacement of synthetic fertilizers with organic amendments). Organic substitution consistently shifted soil pH toward neutrality. Quantitative partitioning revealed that pH amelioration independently accounted for 15.0%, 9.2%, and 4.1% of SOC accrual in acidic, neutral, and alkaline soils, respectively, effects primarily mediated by enhanced microbial biomass. The pH amelioration‐shared SOC accrual underpinned yield gains of 8.6%, 5.1%, and 8.1% across the three pH categories. These benefits were sustained over extended organic substitution durations, with the most pronounced effects in acidic soils. Spatially, pH‐driven yield gains peaked at 9.9% in Africa. Our findings underscore that integrating proactive pH management into organic substitution strategies advances both soil carbon sequestration and global food security.

## Graphical abstract

Soil pH shifts towards neutrality in both acidic and alkaline soils under organic substitution. A global synthesis links pH amelioration to soil organic carbon accrual through enhanced microbial biomass after accounting for added organic carbon and its interaction with pH. This pH amelioration‐shared soil organic carbon accrual accounts for 5.1–8.6% of total yield gains.

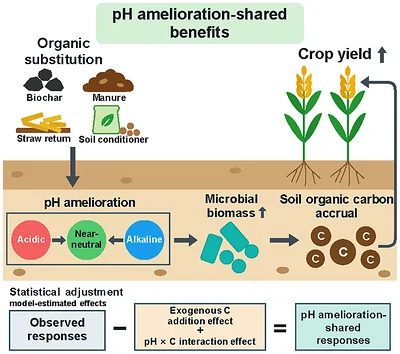

## 1 Introduction

Global food security faces an unprecedented convergence of challenges: the need to increase crop production by at least 35% by 2050 [1], persistent cropland degradation [2], and the imperative to transform croplands from net carbon sources to sinks [3]. Within this trilemma, soil health has emerged as a critical yet underutilized leverage point [4]. Two soil attributes, soil pH and soil organic carbon (SOC), determine the capacity of soil health to resolve this trilemma [5, 6]. SOC stabilizes soil structure, regulates water retention, and drives nutrient cycling [7], while soil pH governs biogeochemical multifunctionality including carbon dynamics and crop productivity, through its regulation of nutrient availability, microbial activity, and ion toxicity [8]. A near‐neutral soil pH optimizes these functions [9], yet anthropogenic pressures have pushed global croplands toward pH extremes, undermining both food production and climate mitigation potential [10]. Intensive agriculture has precipitated a global crisis of concurrent SOC depletion and pH imbalance [11, 12]. Excessive synthetic nitrogen fertilization has triggered widespread acidification, e.g., soils with pH < 5.5 occupy approximately 50% of global croplands [13], causing SOC losses of up to 20% and yield reductions averaging 14% [14]. Concurrently, alkalinization depletes SOC at 3.47 t ha^−^^1^ yr^−^^1^ across approximately 397 million hectares of alkaline croplands [15, 16]. These dual trajectories threaten the yield gains required to meet rising food demand while accelerating soil carbon emissions. Minimizing soil pH deviations from neutrality is therefore a fundamental requirement for sustainable agriculture.

Current pH management strategies present stark trade‐offs. Liming can remediate acidic soils and boost yields in regions such as sub‐Saharan Africa [5], but repeated application degrades soil structure [17] and disrupts microbial communities [18], with uncertain long‐term sustainability. Organic substitution (partial replacement of synthetic fertilizers with organic amendments) offers a nature‐based alternative that simultaneously addresses pH imbalance and carbon sequestration [6]. However, these two outcomes are mechanistically entangled: organic amendments simultaneously alter pH and introduce exogenous carbon, obscuring whether SOC gains arise from pH change per se or simply from carbon addition [19, 20]. Disentangling the pH‐specific contribution to SOC accrual is essential for predictive modeling of soil carbon dynamics and for designing amendment strategies that maximize both food production and climate benefits, yet this remains methodologically unresolved.

This entanglement extends to yield responses. Soil pH governs crop productivity directly, by regulating nutrient availability (e.g., nitrogen) and ion toxicity [21], and indirectly through SOC‐mediated improvements in soil structure and water retention [22, 23]. Under organic substitution, total yield gains reflect the composite effects of exogenous carbon input and soil pH amelioration. Although pH alteration and associated SOC accrual are recognized as the drivers of yield gains [24, 25, 26], their relative contributions have never been quantitatively partitioned. Consequently, the extent to which pH‐mediated SOC sequestration contributes to yield gains and whether this contribution varies across soil types and geographies remain unknown, limiting the evidence base for climate‐smart agricultural policies.

Here, we synthesize data from 251 field trials to isolate the effects of pH amelioration on SOC accrual and yield gains under organic substitution. We employ a weighted mixed‐effects meta‐analytic framework to quantify ‘pH‐mediated SOC accrual’, the SOC increment attributable solely to pH change after controlling for exogenous carbon inputs, and validate these estimates against lime‐only experiments, where pH shifts occur without organic carbon addition. Given that microbial activity is more likely constrained in acidic soils [27] relative to alkaline soils [28], we postulated that SOC accrual upon pH amelioration is relatively greater in acidic soils. This study has three aims: (1) quantifying pH amelioration effects on SOC accrual under organic substitution across acidic, neutral, and alkaline soils; (2) elucidating the mechanisms underpinning SOC responses; and (3) determining the proportion of yield gains attributable to pH amelioration‐shared SOC accrual across global croplands. By resolving these mechanistic linkages across global croplands, this work provides a quantitative foundation for integrating pH management into sustainable intensification strategies and carbon crediting frameworks.

## 2 Results

### 2.1 Soil pH amelioration drives SOC accrual Under organic substitution

Organic substitution consistently shifted soil pH toward neutrality across all initial pH categories. In acidic soils, pH increased by 5.6% (95% confidence interval (CI): 3.7%∼7.6%). Neutral soils exhibited a moderate pH increase of 2.9% (95% CI: 1.3%∼4.5%), while pH decreased by 0.6% in alkaline soils (95% CI: −1.0%∼−0.3%) (Figure 1a). The absolute magnitude of pH change was substantially smaller in alkaline soils than in acidic soils.

**FIGURE 1:**
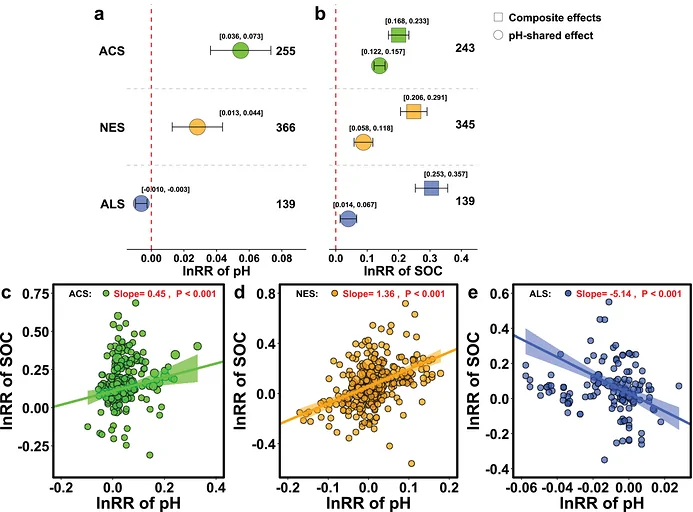
Responses of soil pH (a) and soil organic carbon (SOC) (b) to organic substitution across initial soil pH categories, and the bivariate relationships of pH amelioration‐shared SOC accrual against pH alterations in acidic (c), neutral (d), and alkaline soils (e). In a and b, each dot denotes the weighted mean lnRR, error bars and bracketed values indicate 95% confidence intervals, the number to the right of each dot indicates the number of observations, and red dashed lines mark lnRR = 0. In c–e, the shaded line represents the mean slope and its 95% confidence intervals, with larger point sizes denoting greater weights. lnRR refers to the logarithmic response ratio. In panel b, squares represent the composite effects of organic substitution on SOC, while circles indicate pH amelioration‐shared effects. Colors indicate initial soil pH status: green for acidic soil (ACS), orange for neutral soil (NES), and blue for alkaline soil (ALS).

Total SOC accrued under organic substitution reflected the composite effects of organic carbon addition and pH‐mediated changes. In acidic soils, organic substitution increased SOC by 22.2% (95% CI: 18.2%∼26.2%), of which pH amelioration‐shared SOC accrual can reach up to 15.0% (95% CI: 13.0%∼17.0%) (Figure 1b). The pH‐specific SOC accrual was validated by liming experiments (without organic inputs), which yielded a comparable SOC accrual of 6.8% (95% CI: 1.3%∼12.6%) (Figure S3). In neutral soils, total SOC accrual reached 28.2% (95% CI: 22.9%∼33.7%), with 9.2% (95% CI: 6.0%∼12.5%) driven by pH amelioration (Figure 1b). Alkaline soils showed the largest composite SOC accrual (35.7%, 95% CI: 28.8%∼42.8%), yet the pH amelioration‐shared SOC accrual was modest (4.1%, 95% CI: 1.4%∼6.9%). The significant relationships between SOC accrual and pH amelioration were observed across all initial pH categories. Specifically, SOC accrual increased with rising pH in acidic soils and with declining pH in alkaline soils (Figures 1c, e).

Soil pH amelioration, pH amelioration‐shared SOC accrual, and associated yield gains varied among organic substitution types (Figure S4a–c). Biochar, manure, and soil conditioner increased pH by 2.4% ∼ 9.0% in acidic soils and decreased it by 0.3% ∼ 3.2% in alkaline soils, on average. In contrast, straw substitution did not significantly alter pH in any pH category. Accordingly, straw substitution elicited weaker pH amelioration‐shared SOC accrual relative to the other three substitution types. Regarding yield gains, all substitution types except straw substitution increased yields following pH amelioration in neutral soils (by 8.4% ∼ 26.2%, on average).

These benefits driven by soil pH amelioration were largely sustained over prolonged experimental duration across initial pH categories (Figure S4d–f). At the same period, however, the magnitude of benefits was consistently smaller in alkaline than in acidic soils. Specifically, within the first decade of organic substitution, pH amelioration was 0.6% (95% CI: −0.9%∼−0.3%) in alkaline soils compared to 3.7% (95% CI: 1.7%∼5.7%) in acidic soils. The pH amelioration‐shared SOC accrual was 4.4% (95% CI: 1.3%∼7.5%) in alkaline soils vs. 15.2% (95% CI: 12.8%∼17.6%) in acidic soils; and associated yield gains were 7.5% (95% CI: 2.0%∼13.4%) in alkaline soils compared to 22.3% (95% CI: 20.5%∼24.1%) in acidic soils.

### 2.2 Effects of pH amelioration on soil microbial characteristics and physicochemical properties

pH amelioration‐shared enhancement of microbial biomass was significant in acidic soils (42.2%; 95% CI: 37.6%∼47.0%), alkaline soils (32.9%; 95% CI: 27.9%∼38.0%), and neutral soils (34.8%; 95% CI: 20.7%∼50.6%) under organic substitution (Figure 2a). Lime application (without organic inputs) corroborated this effect, showing a 35.9% (95% CI: 22.2%∼51.2%) increase in microbial biomass (Figure S3). Microbial functionality responses to pH amelioration diverged by initial soil pH categories, with non‐significant change in acidic soils (p = 0.65), a notable increase in alkaline soils (24.3%; 95% CI: 14.7%∼34.6%), and a decline in neutral soils (−60.1%; 95% CI: −75.5%∼−35.1%) (Figure 2a). Bacterial diversity remained unchanged in acidic (p = 0.32) and alkaline soils (p = 0.3), as did fungal diversity in alkaline soils (p = 0.07). In contrast, neutral soils exhibited pronounced alterations, i.e., bacterial diversity increased by 57.8% (95% CI: 41.5%∼75.9%) while fungal diversity reduced by 37.0% (95% CI: −42.8%∼−30.6%) (Figure 2a).

**FIGURE 2:**
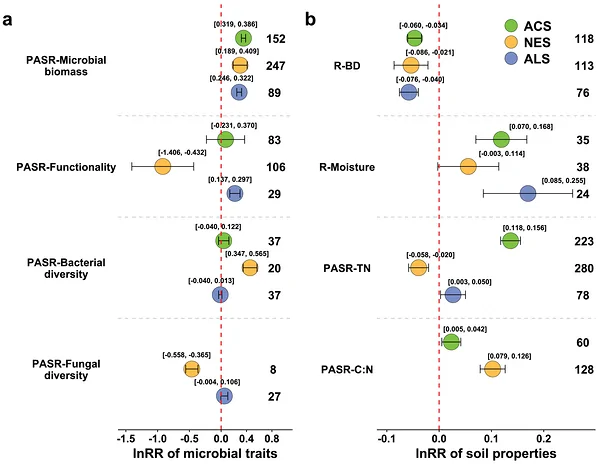
Responses of soil properties across initial soil pH categories, wherein microbial traits, TN, and C:N are pH amelioration‐shared responses (a–b). Each dot represents the weighted mean lnRR, error bars and bracketed values indicate 95% confidence intervals, the number to the right of each dot indicates the number of observations, and red dashed lines mark lnRR = 0. lnRR is the logarithmic response ratio. PASR denotes the response of variables attributable to pH amelioration‐shared effects, and R denotes the response of variables attributable to composite effects under organic substitution. Colors indicate initial soil pH categories: green for acidic soil (ACS), orange for neutral soil (NES), and blue for alkaline soil (ALS). BD, bulk density; TN, total nitrogen; C:N, carbon:nitrogen ratio.

Both soil bulk density and moisture without discriminating pH amelioration‐shared effect changed under organic substitution across initial pH categories. Soil bulk density decreased consistently across all pH categories: acidic (−4.6%; 95% CI: −5.8%∼−3.3%), neutral (−5.2%; 95% CI: −8.3%∼−2.1%), and alkaline soils (−5.6%; 95% CI: −7.3%∼−3.9%). Soil moisture increased most markedly in alkaline soils (18.5%; 95% CI: 8.8%∼29.1%), with a minor rise in neutral soils (5.7%; 95% CI: −0.3%∼12.1%). pH amelioration‐shared accrual of total nitrogen was significant in acidic (14.6%; 95% CI: 12.5%∼16.8%) and alkaline (2.7%; 95% CI: 0.3%∼5.2%) soils, but pH amelioration reduced total nitrogen in neutral soils (−3.8%; 95% CI: −5.7%∼−2.0%). The carbon:nitrogen ratio increased with pH amelioration in acidic (2.4%; 95% CI: 0.5%∼4.2%) and neutral soils (10.8%; 95% CI: 8.2%∼13.5%) (Figure 2b).

### 2.3 Drivers of pH amelioration‐shared SOC accrual

Random forest analysis identified microbial biomass and soil pH as the principal drivers of pH amelioration‐shared SOC accrual, with their relative importance varying by initial soil pH category. Specifically, microbial biomass exerted a stronger influence on pH amelioration‐shared SOC accrual than did pH per se in acidic soils (Figure 3a). Conversely, in neutral and alkaline soils, pH amelioration itself was the dominant driver on pH amelioration‐shared SOC accrual (Figure 3b,c). Climatic factors (mean annual temperature and precipitation) also played important roles in pH amelioration‐shared SOC accrual across all pH categories. Collectively, these drivers explained the vast majority of variation in pH amelioration‐shared SOC accrual under organic substitution, with R^2^ values of 80.0%, 72.4%, and 86.3% in acidic, neutral, and alkaline soils, respectively (Figure 3). SOC accrual was also positively associated with carbon addition rate (Figure S5c).

**FIGURE 3:**
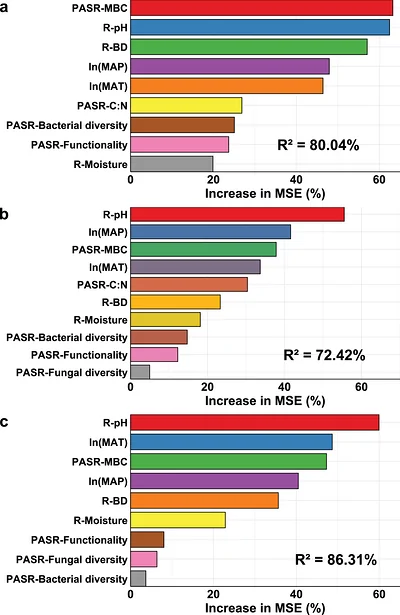
Relative importance of environmental factors in influencing pH amelioration‐shared soil organic carbon (SOC) accrual across initial soil pH categories: acid (a), neutral (b), and alkaline (c). PASR denotes the response of variables attributable to pH amelioration‐shared effects, and R denotes the response of variables attributable to composite effects under organic substitution. ACS, initial acidic soil; NES, initial neutral soil; ALS, initial alkaline soil. MBC, microbial biomass carbon; MAT, mean annual temperature; MAP, mean annual precipitation; BD, bulk density; C:N, carbon:nitrogen ratio.

### 2.4 pH amelioration‐shared SOC accrual contributes to yield gains

Crop yields increased significantly in response to soil pH amelioration. Across soil pH categories, pH amelioration‐shared yield gains were significantly related to both pH amelioration per se and associated SOC accrual (all p < 0.05; Figure S5). Composite yield gains varied by soil pH category, with the highest yield gain by 24.6% in acidic soils followed by 15.3% in neutral soils and 9.5% in alkaline soils (Figure 4a). The role of pH amelioration‐shared SOC accrual in yield gains accounted for a substantial fraction of these gains, namely 8.6% in acidic soils, 5.1% in neutral soils, and 8.1% in alkaline soils (Figure 4b). The yield gains driven by pH amelioration were corroborated by liming trials, in which pH adjustment alone increased yields by 45.4% (Figure S3), confirming the critical role of pH amelioration in yield gains.

**FIGURE 4:**
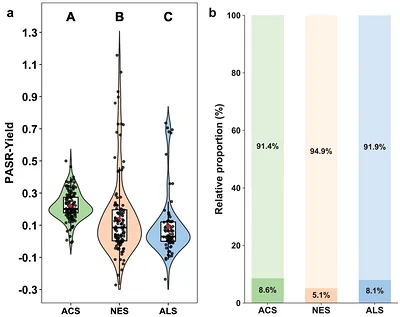
pH amelioration‐shared yield gain across initial soil pH categories (a) and the contribution of pH amelioration‐shared soil organic carbon (SOC) accrual to the total yield gains (b). PASR denotes the response of variables attributable to pH amelioration‐shared effects. In panel a, colored violin envelopes show the kernel density distributions of logarithmic response ratios (lnRR), black dots denote individual observations, and red diamonds denote means for each group. White boxes show the interquartile range (IQR), horizontal lines mark medians, and vertical whiskers follow the 1.5 × IQR rule. Different capital letters denote significant differences between groups (p < 0.05). In panel b, the dark‐shaded lower area denotes the contribution of pH amelioration‐shared SOC accrual to total yield gain. ACS, initial acidic soil; NES, initial neutral soil; ALS, initial alkaline soil.

### 2.5 Mechanistic pathways From pH amelioration to yield gains

Structural equation modeling revealed multiple cascading pathways from soil pH amelioration to yield gains (Figure 5). In acidic soils, pH amelioration significantly affected microbial diversity (standard coefficient = 0.72), biomass (standard coefficient = 0.21), and functionality (standard coefficient = −0.27), with pH amelioration‐shared SOC accrual primarily driven by microbial biomass enhancement, which explained 54.4% of the variance in pH amelioration‐shared SOC accruals (Figure 5a). Neutral and alkaline soils exhibited similar mechanisms, with microbial biomass enhancement following soil pH amelioration significantly supporting pH amelioration‐shared SOC accrual. The efficacy of this microbial biomass effect on pH amelioration‐shared SOC accrual varied across soil pH categories, being strongest in acidic soils (standard coefficient = 0.31) and weaker in neutral (standard coefficient = 0.09) and alkaline soils (standard coefficient = 0.15) (Figure 5).

**FIGURE 5:**
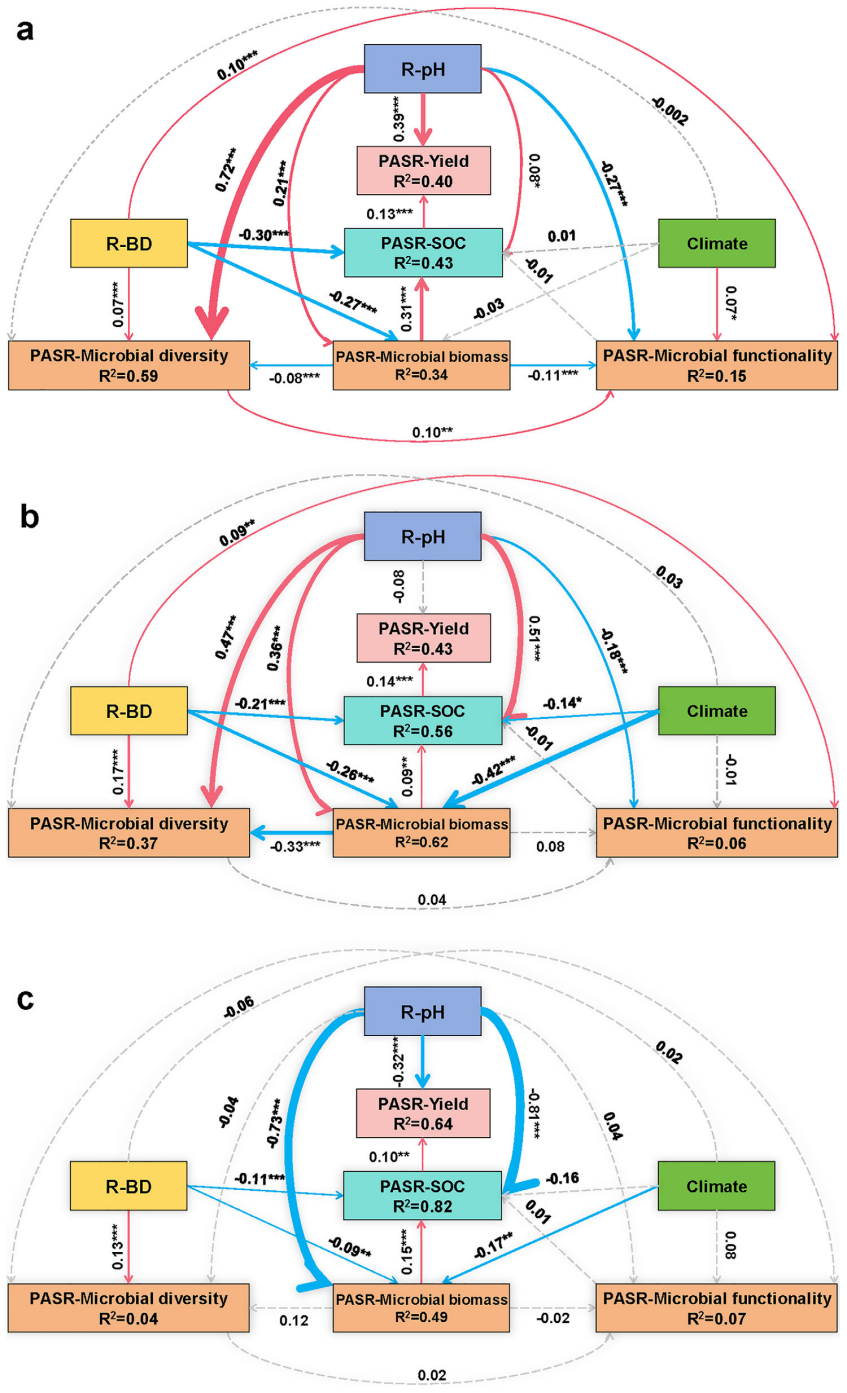
Effects of pH amelioration‐shared environmental and microbial responses on corresponding soil organic carbon (SOC) accrual and yield gains across initial soil pH categories: acid (a), neutral (b), and alkaline (c). Numbers on arrows are standardized coefficients. Red and blue arrows indicate positive and negative relationships, respectively, with thicker arrows indicating stronger effects. PASR denotes the response of variables attributable to pH amelioration‐shared effects, and R denotes the response of variables attributable to composite effects under organic substitution. BD, bulk density. A solid line denotes a statistically significant relationship, and a dashed line denotes a non‐significant relationship. *, p < 0.05; **, p < 0.01; ***, p < 0.001.

A consistent finding across all pH categories was that pH amelioration‐shared SOC accrual significantly contributed to yield gains, with standard coefficients of 0.13, 0.14, and 0.10 for acidic, neutral, and alkaline soil, respectively. Direct pH per se effects on yield gains were also significant in acidic and alkaline soils, but not in neutral soils.

### 2.6 Global patterns of pH amelioration‐shared SOC accrual and associated yield gains

At the global scale, pH amelioration‐shared SOC accrual was widespread under current organic substitution practices (Figure 6a). All continents exhibited significant SOC accrual driven by pH amelioration, with Europe showing the most pronounced enhancement (by 27.4%) and increases on other continents generally exceeding 20% (Figure 6b).

**FIGURE 6:**
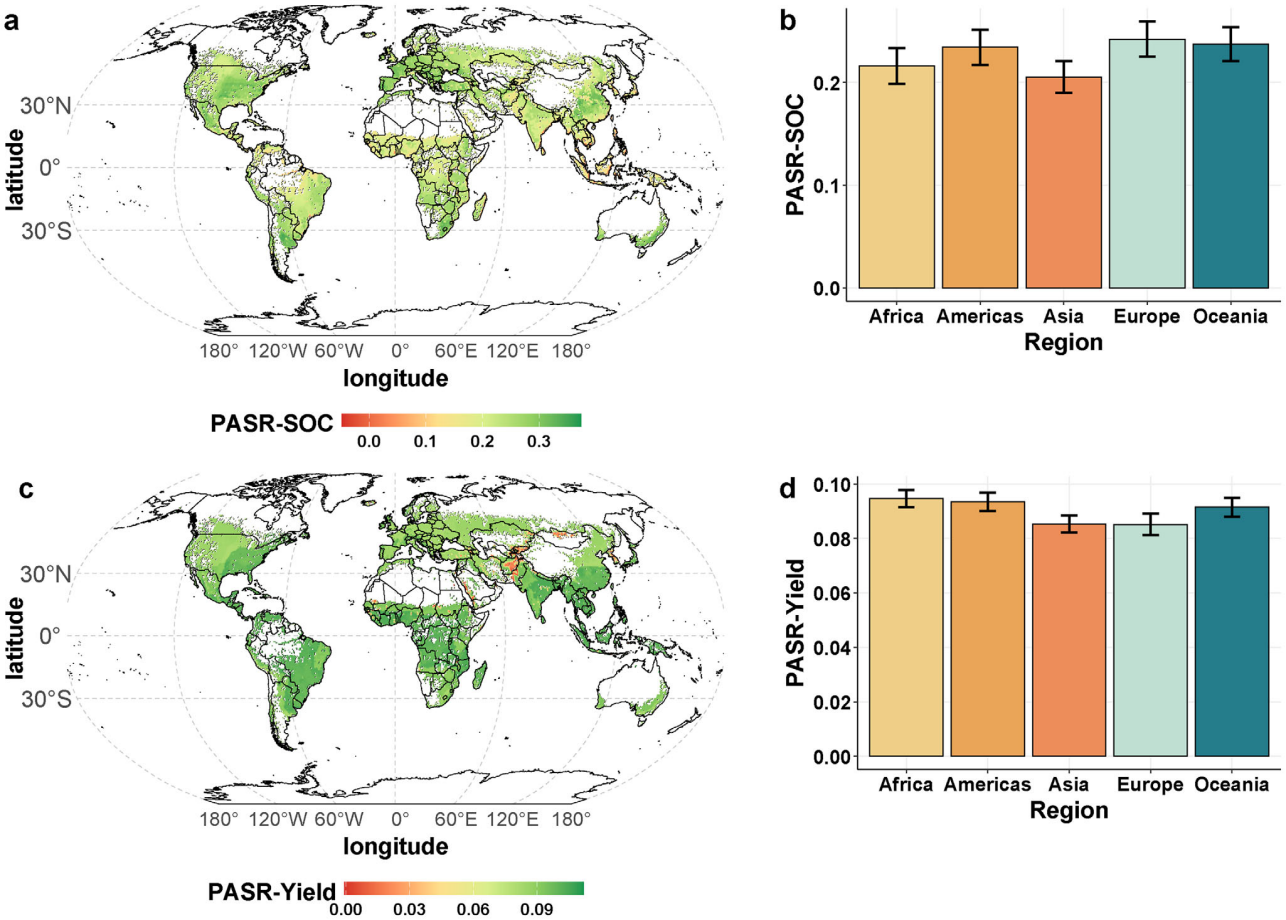
Predicted responses of pH amelioration‐shared soil organic carbon (SOC) accrual (a) and corresponding yield gains (c), and their continental summaries (b and d). PASR denotes the pH amelioration‐shared response, expressed on the natural logarithmic response ratio (lnRR) scale. In panels b and d, error bars denote standard errors.

This pH amelioration‐shared SOC accrual propagated to crop yields worldwide through cascading effects (Figure 6c). Yield gains attributable to pH amelioration were observed across all five continents (Figure 6d), with Africa showing the highest yield gain at 9.9% and approaching 9.0% elsewhere.

## 3 Discussion

Our findings demonstrate that soil pH amelioration under organic substitution initiates a cascading effect: it enhances microbial biomass, which drives SOC accrual and ultimately increases crop yields (Figures 4 and 5). Organic substitution consistently shifts pH toward neutrality, alleviating both acidity and alkalinity extremes (Figure 1), which is different from an earlier viewpoint that straw return exacerbates soil acidification [29]. By quantitatively partitioning the intrinsic role of pH in SOC dynamics, we revealed that pH amelioration per se accounts for up to 15.0% of SOC accrual in acidic soils, with concomitant yield gains (Figures 1b and 4). These results advance the current conceptual framework [30] by empirically isolating pH‐mediated effects from confounding factors. The dual benefits for carbon sequestration (through microbially mediated SOC accrual) and food security (via SOC‐driven yield gains) underscore the transformative potential of targeted pH management in achieving sustainable agriculture.

### 3.1 Mechanisms of pH amelioration‐shared SOC accrual

Soil pH amelioration enhanced SOC accrual through both abiotic and biotic pathways, with the extents varying by initial pH status. In acidic soils, pH amelioration can inhibit acid‐catalyzed hydrolysis [31], slowing the chemical degradation of organic matter components such as polysaccharides and ester bonds and thereby increasing the preservation potential of organic macromolecules [32]. Concurrently, pH amelioration facilitates the deprotonation of carboxyl groups, strengthening their coordination or bridging with polyvalent cations such as Ca^2^^+^ [33] and promoting microaggregate formation via cation bridging [34], which enhances physical protection of SOC within microhabitats [35]. This positive effect was partially offset by reduced positive surface charges on variable‐charge minerals (e.g., Fe and Al oxides), which limited mineral adsorption capacity [36, 37]. In alkaline soils, a slight pH shift toward neutrality can weaken the excessive deprotonation of organic functional groups [38], reduce dissolved organic carbon mobility and colloid dispersion [39], and may prolong organic carbon residence in the solid phase. This process is constrained by the concurrent dissolution of Ca‐bound SOC or carbonate‐associated organic carbon at lower pH, which weakens Ca‐mediated organic carbon preservation [40, 41].

Microbial biomass enhancement following pH amelioration was the primary biotic driver of pH amelioration‐shared SOC accrual (Figure 2a). This effect was corroborated by liming experiments, which showed a 35.9% increase in microbial biomass without organic carbon inputs (Figure S3). Mechanistically, higher microbial biomass increases the extracellular polymeric substance production, which can bind soil minerals through electrostatic interactions and coordination bonds [42, 43]. Microbial residues and cell‐wall components, such as bacterial peptidoglycans and fungal chitin, can contribute recalcitrant carbon to long‐term SOC accrual [44]. Furthermore, microbial polysaccharide secretion and mycelial network formation facilitate soil aggregation [45], restricting microbial and enzymatic access to encapsulated SOC within microenvironments and thereby reducing SOC degradation [46]. Microbially mediated Fe and Mn redox cycling can modify mineral surface properties and enhance organic carbon adsorption capacity [47, 48].

### 3.2 pH amelioration efficacy depending on initial soil pH status

The magnitude of pH amelioration under organic substitution differed markedly between acidic and alkaline soils (Figure 1; Figure S4), reflecting their contrasting buffering capacities. In acidic soils, pH amelioration is achieved through the decarboxylation of organic acids (e.g., malic acid, citric acid) by which H^+^ was consumed [49] and the resultant CO_3_^2−^ can buffer further soil acidification [50]. NH_3_ generation from protein degradation forms NH_4_^+^ and OH^−^ to neutralize H^+^ and raises soil pH [51]. Functional components in organic matter, such as carboxyl and phenolic hydroxyl, can form stable complexes with cations like Al^3^^+^ and Fe^3^^+^, effectively inhibiting the hydrolysis and subsequent acid production [52]. Alkaline cations (e.g., Ca^2^^+^ and Mg^2^^+^) released from organic matter degradation consume H^+^ through ion exchange at soil colloid surfaces, shaping a sustained buffering system [53].

In alkaline soils, neutralization of low‐molecular‐weight organic acids (e.g., citric acid, malic acid, and oxalic acid) with CO_3_^2−^ and OH^−^ reduces alkaline ion concentrations [54]. Proton dissociation from humic acid macromolecules further consumes alkaline ions, enhancing the pH amelioration [55]. Increased cation exchange capacity from negatively charged functional groups (e.g., carboxyl and phenolic hydroxyl) enhances colloid competitiveness for Na^+^ adsorption [56]. Accordingly, Ca^2^^+^ and Mg^2^^+^ released from organic matter decomposition displace Na^+^ around colloid surfaces via ion exchange, reducing the sodium adsorption ratio [57]. CaCO_3_ precipitation provides an additional pathway for soluble alkaline ion removal [58].

The smaller pH amelioration effect in alkaline soils in comparison with acidic soils (Figure 1a) reflects their differentiated buffering capacities. Acidic soils, typically rich in exchangeable H^+^ and acidic metal ions, experience rapid neutralization through organic acids reactions and complexation [59]. In contrast, alkaline soils with their robust carbonate‐bicarbonate buffer system require greater acid inputs and longer durations for significant pH reduction [60]. Nevertheless, the associated 4.1% SOC accrual in alkaline soils was ecologically meaningful (Figure 1a,b). Even a minor pH shift toward neutrality can reduce soluble alkaline ions, weaken Na^+^‐induced dispersion, and improve colloidal stability, thereby favoring organic carbon retention in soil solid phase [61, 62]. This suggests that organic inputs may progressively improve structural stability and fertility in saline‐alkaline croplands by alleviating alkaline constraints.

### 3.3 Yield gains following pH amelioration are driven by cascading effects

Mitigation of soil acidity and alkalinity can significantly enhance crop yields (Figure 4; Figure S4) through optimizing nutrient uptake and regulating soil microbial communities. In acidic soils, Al^3^^+^ and Mn^2^^+^ toxicity competitively inhibit phosphorus and calcium uptake in crops [63]. Raising soil pH from 3.8 to 6.5 increases phosphorus uptake efficiency by 3.7‐fold [64]. In alkaline soils, micronutrients such as zinc form insoluble hydroxides bonded with hydroxyl ions, causing physiological deficiencies in crops [65]. Lowering alkaline soil pH from 8.0 to 7.0 enhances leaf iron concentration by 11%, photosynthetic efficiency by 6%, and biomass by 36% [66].

Moreover, microbial mediation proves equally critical. In general, acidic soils suppress ammonia‐oxidizing microorganism activity, reducing the efficiency of nitrogen transformation and crop yields [67]. The abundance of AOA and AOB increased by 370% and 38%, respectively, when soil pH rose from 4.2 to 6.5, substantially expediting nitrification rate and boosting yields by 21% [68]. Alkaline conditions can impair spore germination [69] and hyphal extension [70] of arbuscular mycorrhizal fungi, reducing phosphorus and zinc uptake in crops via mycelial networks and reducing yields [71]. Neutral soils exhibit 16% greater yield benefits caused by arbuscular mycorrhizal fungi proliferation than alkaline soils [72].

The role of SOC accrual following pH amelioration in promoting crop yields is not ignored (Figure 4). SOC accrual significantly enhances water‐holding capacity [73], with each 10 g kg^−1^ SOC increase raising available water by 1.4 – 1.9 mm·(100 mm)^−^^1^ [74], markedly improving drought resistance [75]. At the global scale, model projections demonstrated that 4.87 GtC SOC accrual in croplands would reduce mean temperature by 0.01°C [75]; given that 1°C – 2°C warming reduces cereal yields by 5% – 16% [76], pH amelioration‐shared SOC accrual partially safeguards crop yield sustainability through both direct soil improvement and indirect climate mitigation. Meanwhile, yield gains may reciprocally enhance SOC accrual by increasing root biomass, rhizodeposition, and crop residue return [77]. Although the reciprocal relationship between yield gains and SOC accrual was not explicitly included in our structural equation models (Figure 5), it likely reinforces long‐term agricultural sustainability.

### 3.4 Continental variation in SOC and yield responses

Both pH amelioration‐shared SOC accrual and associated yield gains were robust across all continents, but their magnitudes varied geographically (Figure 6). The strongest SOC accrual in Europe may reflect the convergence of temperate hydrothermal conditions and conservation‐oriented management. Moderate hydrothermal conditions favor microbial carbon transformation after pH amelioration toward SOC sequestration rather than mineralization [78, 79], consistent with the high explanatory power of climatic factors for pH amelioration‐shared SOC accrual in our random forest analysis (Figure 3). Conservation practices in European croplands, including cover plants, crop rotations, and reduced tillage can further favor pH amelioration‐shared SOC accrual [80]. The most pronounced yield gains in Africa are more likely associated with high marginal benefits of pH amelioration and SOC accrual in low‐fertility croplands. African croplands have long been heavily constrained by nutrient scarcity [81]. Yield potential is further limited by aluminum toxicity that hampers root elongation or development (e.g., sub‐Saharan Africa) [5, 63]. Under such conditions, pH amelioration‐shared SOC accrual substantially improves nutrient availability (e.g., phosphorus) and root activities [5, 82] through enhanced mineralizable nutrient supply, nutrient retention, and water retention [83], and ultimately translates into disproportionately large yield benefits.

### 3.5 Implications and limitations

Our quantitative partitioning reveals that acidic soil regions should be prioritized for pH management in carbon sequestration campaigns, given the 15.0% vs. 4.1% pH amelioration‐shared SOC accrual in acidic vs. alkaline soils (Figure 1b). The substantial yield gains attributable to pH amelioration‐shared SOC accrual (Figure 4b) further demonstrate that pH restoration serves as a cost‐effective strategy for simultaneously advancing food security and carbon neutrality. The microbial mechanisms identified here (Figure 5) highlight a critical gap in current carbon crediting, which predominantly rewards organic inputs while overlooking microhabitat optimization. We advocate for: (1) integrating pH management into climate‐smart agriculture policies, and (2) developing carbon crediting frameworks that recognize soil microhabitat improvement as a valid sequestration pathway.

Several limitations warrant acknowledgment. First, our partitioning estimates, derived from observational meta‐analytic data, provide statistical attribution rather than strict experimental causal inference [84]. Although our model incorporated pH change, exogenous carbon input, their interaction, and between‐study random effects, unmeasured confounders and potential nonlinearities remain unaccounted for. The independent liming dataset provided directional validation, but a global‐scale uniform trial network would be required for definitive causal tracking. Second, soil aggregate stability regulates SOC accrual [85], yet aggregate stability data were insufficient to directly test whether pH amelioration promoted SOC accrual via enhanced physical protection. Third, root trait data (architecture, topology, rhizosphere processes, isotope tracing) were too scarce to partition the contribution of pH‐mediated root responses to nutrient uptake efficiency and yield formation [63], and thus the dynamics of crop roots were not taken into account. Despite these constraints, the coupled responses of pH, SOC, and yield provide foundational evidence for pH amelioration as a lever for sustainable intensification.

## 4 Conclusions

This study provides the first quantitative partitioning of how soil pH amelioration per se drives SOC accrual and cascades to yield gains, revealing a crucial yet underappreciated role of soil microhabitat optimization in global cropland carbon sequestration and food security. Organic substitution, as an effective nature‐based pH remediation strategy, consistently ameliorated soil pH in acidic soils (5.6% increase) and alkaline soils (0.6% decrease). pH amelioration‐shared SOC accrual primarily driven by microbial biomass enhancement accounted for 15.0% in acidic soils, 9.2% in neutral soils, and 4.1% in alkaline soils. These findings identify acidic soil regions as priorities for pH management, where microbial biomass constraints are most alleviated. Yield gains attributable to pH amelioration‐shared SOC accrual accounted for 8.6%, 5.1%, and 8.1% of total yield improvements in acidic, neutral, and alkaline soils, respectively, providing the first empirical evidence of cascading yield benefits from pH amelioration in croplands. These effects remained largely stable over extended organic substitution durations, with the most pronounced benefits in acidic soils. Among organic substitution types, straw return exhibited the weakest efficacy in pH amelioration and corresponding SOC accrual and yield gains. By partitioning the intrinsic contribution of pH amelioration to carbon sequestration and food production, these findings advance the mechanistic understanding of organic amendment effects. The global expectations for SOC accrual and yield gains driven by pH amelioration offer a quantitative basis for integrating proactive soil pH management into climate‐smart agricultural strategies.

## 5 Materials and methods

### 5.1 Data compilation

We conducted a synthesis of peer‐reviewed articles to examine the effects of organic substitution on soil pH and its cascading effects on SOC and yield. Peer‐reviewed articles were collected through the ISI Web of Science and the China National Knowledge Infrastructure (CNKI) databases, up to July 2024, using the following keywords: (“microbial diversity” OR Chao1 OR Ace OR “microbial biomass” OR MBC OR “microbial biomass carbon” OR “carbon cycle enzyme” OR “carbon cycling enzyme”) AND (SOC OR “Soil Organic Carbon” OR yield OR “crop production”) AND (“Organic fertiliz*”). This search initially identified 2775 records, of which 84 publications were retained after duplicate removal, abstract screening, and full‐text eligibility assessment (Figure S1a).

Furthermore, we compiled a separate auxiliary dataset from lime application experiments without exogenous organic carbon inputs. This dataset was used to independently calculate the pH amelioration‐shared responses, which served as an independent benchmark for the estimates derived from the organic substitution dataset. For this purpose, a targeted search was performed using the following keywords: (“microbial diversity” OR Chao1 OR Ace OR “microbial biomass” OR MBC OR “microbial biomass carbon” OR “carbon cycle enzyme” OR “carbon cycling enzyme”) AND (SOC OR “Soil Organic Carbon” OR yield OR “crop production” OR pH) AND (“lime” OR “liming”). The studies included in this dataset featured lime application as the sole treatment variable, thus enabling a direct assessment of the responses driven purely by soil pH change. This targeted search initially identified 519 records, of which 15 publications were retained after the same screening procedure (Figure S1b).

The article screening followed PRISMA guidelines, with the procedure shown in Figure S1. Peer‐reviewed articles were screened according to the following criteria: (1) The articles reported changes in soil pH and SOC, and clearly recorded the types and amounts of organic fertilizers applied (including organic input amount). (2) The studies were conducted under field conditions, and only field trials covering at least one complete crop growing season from sowing or transplanting to crop harvest were retained [86]. (3) The articles contained sufficient statistical data, such as means, standard deviations, and sample sizes.

Following screening, 251 experimental studies on organic substitution were eligible. In parallel, a second dataset comprising 29 experimental studies on lime application was compiled for validation. Data were extracted from these studies, encompassing SOC, pH, organic carbon input rates, environmental variables, and microbial characteristics. In addition, experimental duration and amendment type were extracted. Experimental duration refers to the period between the initiation of field treatment and soil sampling, and was standardized in years. Observations were subsequently grouped into short‐term (< 10 years), medium‐term (10–20 years), and long‐term (> 20 years) categories [6]. Organic substitution was defined as the partial replacement of synthetic fertilizers with organic amendments in paired field comparisons, and treatments were further classified into four amendment categories, including biochar, manure, straw return, and soil conditioner [87]. The GetData software (version 2.22) was employed to digitize data from graphical sources, while some datasets were provided directly by the authors in the form of tables or Supplementary Materials. The response of variables was calculated based on paired treatments. Additionally, geographic data (latitude and longitude), mean annual temperature (MAT), mean annual precipitation (MAP), pH alteration rate, and nitrogen addition rate were also compiled from each experimental site. The spatial distribution of the experimental sites and their corresponding climatic factors are shown in Figure S2. To enhance the understanding of how soil pH alterations upon organic substitution impact microbial diversity, biomass, functionality, and carbon accrual, we binned the data into three initial soil pH categories: acidic soils (initial pH < 6), neutral soils (6 < initial pH < 8), and alkaline soils (initial pH > 8) [88].

### 5.2 Data analyses

In each case study, the changes in soil pH under organic substitution and their effects on microbial characteristics and carbon accrual were quantified using natural logarithm of response ratio (lnRR) [89]. An lnRR value of zero indicates no significant effect of soil changes on measured variable, while positive and negative lnRR values respectively signify increases and decreases in the measured variables. Calculations of lnRR and its variance (*v_i_*) were performed according to Equation (1) and Equation (2).

(1) $$\ln\;\mathrm{RR}=\ln\left(X_{t}/X_{c}\right)=\ln\left(X_{t}\right)-\ln\left(X_{c}\right)$$

(2) $$v_{i}=\frac{S_{t}^{2}}{N_{t}X_{t}^{2}}+\frac{S_{c}^{2}}{N_{c}X_{c}^{2}}$$

where, *X_t_* and *X_c_* represent the mean values of the treatments under organic substitution and the control treatment, respectively. Similarly, *N_t_* and *N_c_* are the sample sizes of the organic substitution and control treatments, respectively, while *S_t_* and *S_c_* are their corresponding standard deviations. Additionally, *i* denotes the *i_th_* observation.

Microbial functionality in this study was characterized by the activity of four key enzymes involved in the soil carbon cycling: *α*‐1,4‐glucosidase, *β*‐1,4‐glucosidase, cellulase, and invertase [90].

A mixed‐effects meta‐analysis was conducted using the *‘metafor’* package in R (version 4.4.2) to examine the effects of organic substitution on SOC, soil properties, and microbial characteristics. We adjusted for correlations among multiple comparisons within studies using a covariance matrix [91]. A hierarchical model with nested random effects (∼1 | study/ID) was employed to account for between‐ and within‐study variability. The restricted maximum likelihood method was used for parameter estimation.

To assess publication bias for each variable, we employed Egger's test and the fail‐safe number method. The results of Egger's test were obtained using the *‘metafor’* package (Table S1). Although some variables exhibited slight publication bias, their fail‐safe numbers were all substantially greater than 5n + 10 (n is the number of observations; Table S1), indicating that the impact of publication bias was negligible overall.

For the ‘lime application’ dataset, which was compiled separately for validation purposes, we employed an identical analytical workflow, including the same procedures for effect size calculation, weighted mixed‐effects meta‐analysis (Figure S3), and publication bias assessment (Table S1), to ensure that the results were directly comparable with those of the main dataset.

The pooled lnRR for each variable was converted to a percentage change as follows:

(3) $$\mathrm{Chan}\mathrm{ge}\left(\%\right)=\left(e^{\ln\;\mathrm{RR}}-1\right)\times 100\%$$

A weighted mixed‐effects model was performed on the organic substitution dataset, with soil pH alterations, exogenous organic carbon inputs, and their interaction included as fixed effects [92]. The effects of exogenous organic carbon input and its interaction with soil pH alterations were removed from the total response in Equation (5). The remaining effect size, represented as *Y*_1_, is the pH amelioration‐shared response. To account for between‐study heterogeneity, individual studies were treated as a random effect, and each observation was weighted by the inverse of its variance in the weighted mixed‐effects model.

(4) $$Y=\alpha_{1}\times X_{1}+\alpha_{2}\times X_{2}+\alpha_{3}X_{1}\times X_{2}+\pi_{study}+\varepsilon$$

(5) $$Y_{1}=Y-\alpha_{2}\times X_{2}-\alpha_{3}X_{1}\times X_{2}$$

where *Y* denotes the total response for a variable, and *Y*_1_ denotes its pH amelioration‐shared response. *X_1_* and *X_2_* are the pH changes and exogenous organic carbon input amount, respectively. Moreover, *α_1_*, *α_2_*, *α_3_*, *π_study_*, and *ε* represent the coefficient of pH changes and exogenous organic carbon input amount, the coefficient of the interaction between pH changes and exogenous organic carbon input amount, the random effect of ‘study’, and sampling error, respectively.

Bulk density and soil moisture were reported as composite responses to organic substitution rather than pH amelioration‐shared effects, because both variables are strongly controlled by factors (e.g., soil texture and tillage practices) rather than pH [93].

To further examine whether pH amelioration‐shared effects on SOC accrual and yield gains differed with experimental duration and organic substitution type, subgroup meta‐analyses were conducted using the same analytical framework (Equations (1)–(5)). These subgroup analyses were performed separately for acidic, neutral, and alkaline soils, and only subgroups with sufficient observations (n > 4) were retained.

The bivariate relationship between the response of SOC and soil pH changes was tested using weighted linear mixed‐effects models [92]. In this weighted mixed‐effects model, the inverse of the variance of lnRR between pairs was treated as the weight, and different experimental cases were considered as random effects. The equation is as follows:

(6) $$Z=\beta_{0}+\beta_{1}\times X+\pi_{study}+\varepsilon$$

where *Z* is the response of SOC and *X* represents the soil pH changes, *β*_0_ and *β*_1_ are the intercept and slope values of the model, respectively.

This study employed the ‘*rfPermute*’ package in R in combination with the random forest algorithm and permutation testing, to systematically evaluate the effects of various predictor variables on SOC variation across soils with different initial pH categories (i.e., acidic, neutral, and alkaline).

To evaluate differences in yield response among groups, one‐way analysis of variance (ANOVA) was performed using the *‘stats’* package in R, followed by post‐hoc Least Significant Difference (LSD) tests with the *‘agricolae’* package to identify significant group differences.

Structural equation models (SEMs) were employed to investigate the multivariate relationships among pH amelioration, associated SOC accrual, microbial traits, yield gains, and other environmental factors. Based on the theoretical framework of ‘pH amelioration‐microbial traits‐carbon cycling‐yield’, we specified a pipeline starting from pH amelioration and ending with yield gains. This pipeline followed previous meta‐analytical work that used path modeling to evaluate the relationships among fertilization management, SOC change, and crop production [94]. Specifically, pH amelioration, associated changes in microbial traits, and soil physicochemical properties generally represent earlier soil environmental responses following organic substitution, while pH amelioration‐shared SOC accrual represents the accumulated response along this pipeline, and yield gains represent the end outcome.

In this model, environmental factors, including pH amelioration, climatic conditions, and soil physicochemical properties, serve as exogenous variables that directly or indirectly affect pH amelioration‐shared responses of microbial diversity, biomass, and functionality. These microbial traits further regulate SOC accrual, which subsequently influences yield gains following pH amelioration. Based on the initial soil pH conditions, including acidic, neutral, and alkaline soils, three separate SEMs were constructed. The models hypothesized that pH amelioration per se influences SOC accrual through dual pathways, including direct alteration of the soil chemical environment and indirect regulation of microbial traits.

During the model construction, all potential causal variables were initially included, followed by removal of redundant variables to simplify the models. Model selection was based on minimizing the Akaike Information Criterion (AIC) and retaining models with acceptable goodness of fit based on Fisher's C tests. The final piecewise SEMs showed adequate fit for acidic, neutral, and alkaline initial soil pH groups, with Fisher's C values of 9.598, 11.820, and 15.829, respectively. Each model had 12 degrees of freedom, with corresponding *p* values of 0.650, 0.460, and 0.199. All SEM analyses were conducted using the *‘piecewiseSEM’* package in R.

A machine learning approach was employed to predict the global changes of pH amelioration‐shared SOC accrual and its cascading effect on yield gains. The XGBoost algorithm modeled the response variables (i.e., the lnRR of pH amelioration‐shared SOC accrual and associated yield gains) based on a suite of gridded predictors. To ensure spatial consistency, all predictors were resampled to a 0.5° × 0.5° resolution. To achieve predictive robustness, a 100‐iteration ensemble approach was implemented, whereby in each iteration the dataset was randomly partitioned into a 70% training set and a 30% validation set. Model performance was evaluated using the coefficient of determination (R^2^), root mean square error (RMSE), and mean absolute error (MAE). The models demonstrated robust predictive accuracy, achieving high R^2^ values and low prediction errors for both the SOC (R^2^ = 0.71, RMSE = 0.09, MAE = 0.07) and yield (R^2^ = 0.77, RMSE = 0.01, MAE = 0.01) responses, with predicted values aligning closely with observed values along the 1:1 line (Figure S6). We generated the final global distribution maps through pixel‐wise averaging of the 100 model outputs, which were then aligned by a global cropland layer to precisely delineate effects within agricultural ecosystems. The continental‐scale assessment was binned into five regions (i.e., Africa, Americas, Asia, Europe, and Oceania) using the ‘*rnaturalearth*’ R package, after which zonal statistics were computed to quantify inter‐regional variations.

## Author Contributions

ZL conceived the research. XD collected and analyzed the data. XD and ZL wrote the first manuscript. ZL heavily revised the manuscript. All authors contributed to the review of the manuscript.

## Conflicts of Interest

The authors declare no conflicts of interest.

## Supporting information




**Supporting File**: advs78051‐sup‐0001‐SuppMat.docx.

## Data Availability

The dataset generated in this study is archived in the GitHub repository and is freely available at https://github.com/Alice5412/data2‐ph‐soc.git.
