# Supplementary material for: Soil pH Amelioration Synergizes Carbon Accrual with Yield Gains Under Organic Substitution
Source: Adv Sci (Weinh). 2026 Sep 28:e78051. Online ahead of print. doi: 10.1002/advs.78051 (PMC13618767; doi:10.1002/advs.78051)
Supplement: Supplementary file 1 [file ADVS-9999-e78051-s001.docx]

**Table S1.** Results of publication bias assessment using Egger’s test and fail-safe-number. PASR denotes the response of variables attributable to pH amelioration-shared effects, whereas R denotes the response of variables attributable to composite effects under organic substitution. ACS, acidic soils; NES, neutral soils, ALS, alkaline soils. SOC, soil organic carbon; C:N, carbon to nitrogen ratio; TN, total nitrogen; BD, bulk density. n, number of observations. BA, biochar; MA, manure; SR, straw return; SC, soil conditioner. Experimental durations are classified as short-term (<10 years), medium-term (10–20 years), and long-term (>20 years).

| **Groups** | **Variable** | **n** | **p** | **Fail-safe number** |
| --- | --- | --- | --- | --- |
| ACS | R-pH | 255 | 0.6533 |  |
|  | PASR-SOC | 243 | P < 0.0001 | 301549 > 5n+10 |
|  | R-SOC | 243 | P < 0.0001 | 354556 > 5n+10 |
|  | PASR-C:N | 60 | P < 0.05 | 707 > 5n+10 |
|  | PASR-TN | 223 | P < 0.0001 | 173575 > 5n+10 |
|  | R-BD | 118 | 0.1622 |  |
|  | R-Moisture | 35 | P < 0.05 | 18949 > 5n+10 |
|  | PASR-Microbial functionality | 83 | P < 0.05 | 278433 > 5n+10 |
|  | PASR-Microbial biomass | 152 | 0.269 |  |
|  | PASR-Bacterial diversity | 37 | 0.9233 |  |
| NES | R-pH | 366 | 0.0738 |  |
|  | PASR-SOC | 345 | 0.4192 |  |
|  | R-SOC | 345 | 0.9178 |  |
|  | PASR-C:N | 128 | 0.8311 |  |
|  | PASR-TN | 280 | 0.7629 |  |
|  | R-BD | 113 | 0.2439 |  |
|  | R-Moisture | 38 | P < 0.05 | 213 > 5n+10 |
|  | PASR-Microbial biomass | 247 | P < 0.0001 | 6235190 > 5n+10 |
|  | PASR-Microbial functionality | 106 | 0.1008 |  |
|  | PASR-Bacterial diversity | 20 | 0.8815 |  |
|  | PASR-Fungal diversity | 8 | 0.6552 |  |
| ALS | R-pH | 139 | 0.921 |  |
|  | PASR-SOC | 139 | 0.1621 |  |
|  | R-SOC | 139 | P < 0.05 | 797945 > 5n+10 |
|  | PASR-TN | 78 | 0.0583 |  |
|  | R-BD | 76 | 0.976 |  |
|  | R-Moisture | 24 | 0.7914 |  |
|  | PASR-Microbial biomass | 89 | 0.1903 |  |
|  | PASR-Microbial functionality | 29 | 0.2548 |  |
|  | PASR-Bacterial diversity | 37 | 0.0587 |  |
|  | PASR-Fungal diversity | 27 | 0.0529 |  |
| Lime addition | pH | 110 | P < 0.0001 | 149847 > 5n+10 |
|  | SOC | 54 | 0.3198 |  |
|  | C:N | 14 | P < 0.001 | 130 > 5n+10 |
|  | Microbial biomass | 86 | 0.0021 | 81265 > 5n+10 |
|  | Microbial functionality | 41 | 0.2953 |  |
|  | Bacterial diversity | 9 | 0.3885 |  |
|  | Yield | 32 | P < 0.05 | 81265 > 5n+10 |
| BA ACS | R-pH | 78 | P < 0.05 | 870 > 5n+10 |
|  | PASR-SOC | 76 | 0.1817 |  |
|  | PASR-Yield | 21 | 0.9504 |  |
| BA NES | R-pH | 109 | 0.404 |  |
|  | PASR-SOC | 101 | 0.1688 |  |
|  | PASR-Yield | 55 | 0.826 |  |
| BA ALS | R-pH | 45 | 0.7383 |  |
|  | PASR-SOC | 45 | 0.4434 |  |
|  | PASR-Yield | 21 | 0.2039 |  |
| MA ACS | R-pH | 103 | P < 0.05 | 19040 > 5n+10 |
|  | PASR-SOC | 95 | P < 0.0001 | 34877 > 5n+10 |
|  | PASR-Yield | 59 | 0.3834 |  |
| MA NES | R-pH | 184 | 0.0791 |  |
|  | PASR-SOC | 182 | 0.9036 |  |
|  | PASR-Yield | 48 | 0.2099 |  |
| MA ALS | R-pH | 67 | P < 0.05 | 424 > 5n+10 |
|  | PASR-SOC | 67 | 0.5364 |  |
|  | PASR-Yield | 49 | 0.1583 |  |
| SR ACS | R-pH | 63 | 0.683 |  |
|  | PASR-SOC | 61 | 0.2036 |  |
|  | PASR-Yield | 47 | P < 0.001 | 13994 > 5n+10 |
| SR NES | R-pH | 41 | P < 0.05 | 266 > 5n+10 |
|  | PASR-SOC | 31 | 0.5861 |  |
|  | PASR-Yield | 11 | 0.1609 |  |
| SR ALS | R-pH | 16 | 0.8875 |  |
|  | PASR-SOC | 16 | 0.265 |  |
| SC ACS | R-pH | 11 | 0.2311 |  |
|  | PASR-SOC | 11 | 0.3664 |  |
|  | PASR-Yield | 7 | 0.8492 |  |
| SC NES | R-pH | 32 | P < 0.05 | 11391 > 5n+10 |
|  | PASR-SOC | 31 | P < 0.05 | 8573 > 5n+10 |
|  | PASR-Yield | 8 | P < 0.0001 | 60 > 5n+10 |
| SC ALS | R-pH | 11 | 0.7158 |  |
|  | PASR-SOC | 11 | 0.6814 |  |
| Short-term ACS | R-pH | 200 | P < 0.0001 | 29284 > 5n+10 |
|  | PASR-SOC | 192 | P < 0.05 | 202261 > 5n+10 |
|  | PASR-Yield | 113 | 0.3559 |  |
| Short-term NES | R-pH | 249 | P < 0.0001 | 48094 > 5n+10 |
|  | PASR-SOC | 233 | 0.3918 |  |
|  | PASR-Yield | 113 | 0.5043 |  |
| Short-term ALS | R-pH | 121 | P < 0.0001 | 1380 > 5n+10 |
|  | PASR-SOC | 121 | P < 0.05 | 56759 > 5n+10 |
|  | PASR-Yield | 70 | P < 0.05 | 1493 > 5n+10 |
| Medium-term ACS | R-pH | 17 | 0.8577 |  |
|  | PASR-SOC | 15 | P < 0.05 | 2446 > 5n+10 |
| Medium-term ALS | R-pH | 11 | 0.4975 |  |
|  | PASR-SOC | 11 | 0.6814 |  |
|  | PASR-Yield | 6 | 0.8216 |  |
| Long-term ACS | R-pH | 38 | 0.7362 |  |
|  | PASR-SOC | 36 | 0.2299 |  |
|  | PASR-Yield | 18 | 0.4352 |  |
| Long-term NES | R-pH | 116 | 0.4341 |  |
|  | PASR-SOC | 111 | 0.5507 |  |
|  | PASR-Yield | 9 | 0.4689 |  |
| Long-term ALS | R-pH | 7 | 0.8725 |  |
|  | PASR-SOC | 7 | 0.8725 |  |

**Figure S1.** PRISMA flow diagrams illustrating the literature selection process for the organic substitution dataset (a) and the liming dataset (b).

**Figure S2.** Distribution of experimental sites in this study. Inset scatter plot shows the profiles of climatic factors. MAT, mean annual temperature; MAP, mean annual precipitation.

**Figure S3.** Responses of soil properties and crop yield to lime addition. Points represent weighted mean natural logarithmic response ratios (lnRR), with error bars and bracketed values indicating 95% confidence intervals. Numbers to the right indicate the number of observations. The red dashed line marks lnRR = 0. SOC, soil organic carbon; C:N, carbon:nitrogen ratio; MBC, microbial biomass carbon.

**Figure S4.** Comparisons of soil pH alteration, pH amelioration-shared soil organic carbon (SOC) accrual, and pH amelioration-shared yield gains among organic substitution types (a–c) and experimental durations (d–f). Dot with an error bar denotes the weighted mean lnRR and its 95% confidence interval. Values in brackets next to each dot are the 95% confidence interval, and the number to the right is the observation number for each subgroup. Colors distinguish initial soil pH categories: green for acidic soils (ACS), orange for neutral soils (NES), and blue for alkaline soils (ALS). The red dashed line is lnRR = 0. BA, biochar; MA, manure; SR, straw return; SC, soil conditioner. lnRR, natural logarithmic response ratio.

**Figure S5.** Bivariate relationships of pH amelioration-shared yield gains against soil pH alteration (a) and pH amelioration-shared soil organic carbon (SOC) accrual (b), and of pH amelioration-shared SOC accrual against organic carbon addition (c) across initial soil pH categories. Solid lines represent mean slopes and shaded areas indicate 95% confidence intervals for acidic (green), neutral (orange), and alkaline (blue) soils, respectively, with larger point sizes denoting greater weights. Slopes and P values are shown in each panel. lnRR, natural logarithmic response ratio. ACS, acidic soils; NES, neutral soils; ALS, alkaline soils.

**Figure S6.** Comparison of predicted versus observed logarithmic response ratios (lnRR) for soil organic carbon (SOC) accrual (a) and yield gains (b). The solid green line is the 1:1 line, the red dashed line is the regression line, and each circle represents an individual observation. R^2^, coefficient of determination; RMSE, root mean square error; MAE, mean absolute error.
